# A Mediterranean Diet with Fresh, Lean Pork Improves Processing Speed and Mood: Cognitive Findings from the MedPork Randomised Controlled Trial

**DOI:** 10.3390/nu11071521

**Published:** 2019-07-04

**Authors:** Alexandra T. Wade, Courtney R. Davis, Kathryn A. Dyer, Jonathan M. Hodgson, Richard J. Woodman, Hannah A. D. Keage, Karen J. Murphy

**Affiliations:** 1 Alliance for Research in Exercise, Nutrition and Activity, School of Health Sciences, University of South Australia, GPO Box 2471, 5001 Adelaide, Australia; 2 School of Medical and Health Sciences, Edith Cowan University, Perth, WA 6027, Australia; 3 Medical School, University of Western Australia, 35 Stirling Highway, Perth, WA 6000, Australia; 4 Flinders Centre for Epidemiology and Biostatistics, Flinders University, GPO Box 2100, 5042 Adelaide, Australia; 5 Cognitive Ageing and Impairment Neurosciences, University of South Australia, GPO Box 2471, 5005 Adelaide, Australia; 6 School of Pharmacy and Medical Sciences, University of South Australia, GPO Box 2471, 5005 Adelaide, Australia

**Keywords:** MedDiet, protein, cognitive function, ageing, cardiovascular disease

## Abstract

Abstract: Background: The Mediterranean diet may be capable of improving cognitive function. However, the red meat restrictions of the diet could impact long-term adherence in Western populations. The current study therefore examined the cognitive effects of a Mediterranean diet with additional red meat. Methods: A 24-week parallel crossover design compared a Mediterranean diet with 2–3 weekly servings of fresh, lean pork (MedPork) and a low-fat (LF) control diet. Thirty-five participants aged between 45 and 80 years and at risk of cardiovascular disease followed each intervention for 8 weeks, separated by an 8-week washout period. Cognitive function was assessed using the Cambridge Neuropsychological Test Automated Battery. Psychological well-being was measured through the SF-36 Health Survey and mood was measured using the Profile of Mood States (POMS). Results: During the MedPork intervention, participants consumed an average of 3 weekly servings of fresh pork. Compared to LF, the MedPork intervention led to higher processing speed performance (*p* = 0.01) and emotional role functioning (*p* = 0.03). No other significant differences were observed between diets. Conclusion: Our findings indicate that a Mediterranean diet inclusive of fresh, lean pork can be adhered to by an older non-Mediterranean population while leading to positive cognitive outcomes.

## 1. Introduction

Dementia is characterised by severe cognitive impairment and reduced quality of life. As the population ages, the prevalence of age-related disorders like dementia is predicted to increase [1]. However, recent reports indicate that 30–35% of dementias may be preventable through risk factor modification [2]. 

Modifiable risk factors for dementia include hypertension, obesity, diabetes, smoking, and physical activity. Multi-domain interventions with the ability to improve health across these risk factors are now being investigated for their potential to delay cognitive decline and reduce risk of dementia [3]. 

The Mediterranean diet is a predominantly plant-based diet, characterised by the high consumption of extra virgin olive oil, vegetables, fruits, nuts, seeds and whole grains; a moderate intake of fish, poultry and dairy foods; and a low intake of red meat and processed foods [4]. The Mediterranean diet is therefore rich in bioactive nutrients, including mono and polyunsaturated fatty acids, polyphenols, flavanols, carotenoids, essential vitamins and fibre. Observational studies have demonstrated that populations following a traditional Mediterranean diet experience less cognitive decline and a lowered risk of dementia [5,6]. Similarly, clinical trials have reported that participants following a Mediterranean dietary pattern achieve higher performance on tests sensitive to ageing and dementia compared to those following a low-fat control diet [7,8,9]. 

The cognitive benefits of the Mediterranean diet may be due to improvements in cardiovascular health. Clinical studies have shown that the Mediterranean diet is capable of reducing blood pressure, inflammation and atherosclerosis [10,11]. By improving systemic blood flow, the Mediterranean diet may be capable of increasing blood and oxygen supply to the brain, and preventing premature brain cell death [12]. Additionally, nutrients consumed within a Mediterranean diet may have direct benefits for brain cell function. Omega-3 polyunsaturated fatty acids are a vital component of neural membrane growth, repair, structure and function [13]. Further, omega-3 fatty acids produce anti-inflammatory lipid mediators in the brain, while polyphenols and flavanols may have anti-oxidant effects and protect against oxidative stress. Omega-3 fatty acids are unable to be synthesised by the body and must be obtained from the diet. By providing rich sources of omega-3 fatty acids such as oily fish, walnuts and seeds, the Mediterranean diet may influence brain cell function, integrity and health [12].

The Mediterranean diet may be an appropriate lifestyle intervention in Australia, where dementia is one of the leading causes of disability, the second leading cause of death, and the leading cause of death in women [14]. Literature investigating the palatability of Mediterranean diets in non-Mediterranean populations is relatively nascent. However, our research group recently reported that the red meat restrictions of the Mediterranean diet were one of the biggest challenges to sustaining adherence [15]. A Mediterranean diet that allows additional red meat sources may therefore improve feasibility and palatability in non-Mediterranean populations. Red meat supplementation may also provide additional cognitive benefits to a Mediterranean dietary pattern. Protein-rich foods, such as lean red meat, provide creatine and thiamine. Creatinine and thiamine are involved in brain energy metabolism and homeostasis, and may influence cognitive function [16,17,18]. Further, diets higher in protein have been linked to a reduced risk of dementia [16]. 

In Australia, beef and pork are the most frequently consumed sources of red meat [19]. In comparison to beef, fresh, lean pork contains less heme iron and less saturated fat per 100 g. Pork may then be a suitable addition to the Mediterranean diet, which is traditionally low in heme iron sources and saturated fat. Previous research also indicates that chicken can be replaced with pork without negative effects on blood lipids, glucose, insulin or cognitive function [20,21]. Further, pork production has been associated with significantly less greenhouse gas emissions compared with ruminant meats [22]. The inclusion of pork will therefore have less of an impact on the environmental sustainability of the dietary pattern. 

As reported elsewhere, a Mediterranean diet supplemented with fresh, lean pork is comparable to a low-fat diet for cardiovascular outcomes [23]. However, the cognitive effects of the diet are unknown. The current study therefore examined a Mediterranean diet modified to include 2–3 fresh servings of pork each week across measures of cognitive function and well-being. In line with previous cardiovascular and cognitive research [7,24,25], a low-fat control was employed. It was hypothesised that the modified Mediterranean diet would lead to greater improvements in cognitive functions associated with ageing and dementia compared with the control diet.

## 2. Methods 

The MedPork trial was designed to evaluate the cardiovascular and cognitive effects of a Mediterranean diet supplemented with fresh, lean pork. The study protocol and cardiovascular findings have been published elsewhere [23,26]. The secondary cognitive and psychological outcomes are presented herein. 

### 2.1. Ethics

This trial was registered with the Australian New Zealand Clinical Trials Registry (ACTRN12616001046493) on 5 August, 2016 and was conducted in accordance with the Declaration of Helsinki. All procedures involving human participants were approved by the University of South Australia Human Ethics Committee (#35662). This trial was registered with the Australian New Zealand Clinical Trials Registry (ACTRN12616001046493) on 5th August 2016.

### 2.2. Participants and Recruitment

Volunteers were recruited from metropolitan Adelaide via electronic and paper advertisements. Adults aged 45–80 years were recruited due to their increased risk of developing cardiovascular disease (CVD) [27]. Eligible volunteers were required to have elevated systolic blood pressure (≥120 mmHg) and at least two of the following cardiovascular risk factors: Body mass index ≥ 25 kg/m^2^; elevated fasting total cholesterol (≥5.5 mmol/L), triglycerides (≥2.0 mmol/L), low-density lipoprotein (≥3.5mmol/L), or low levels of high-density lipoprotein (≤0.9 mmol/L for men and ≤1.0 mmol/L for women); elevated fasting glucose (between 6.1 and 7.8 mmol/L); and/or a family history (up to one generation) of CVD or type 2 diabetes mellitus. Volunteers were excluded if they met any of the following criteria: Currently taking antihypertensive medication; current smoker; current CVD or angina; current or recent (within 6 months) malignancies; respiratory disease; gastrointestinal disease; kidney disease; type 2 diabetes mellitus; a current or previous traumatic head or brain injury; a current neurological or psychiatric condition; antidepressant or anxiety medication; a current diagnosis of Alzheimer’s disease or dementia; currently following a Mediterranean-style diet, as assessed by a score of 10 or above on the Prevencionn con Dieta Mediterranea (PREDIMED) 14-point checklist [24], or currently consuming supplemental omega-3 fatty acids >1000mg daily. Other dietary supplements were permitted on the condition that they had been consumed for three months prior to screening and were consumed consistently throughout the trial.

Eligibility was determined using a diet and lifestyle questionnaire and follow-up screening visit at the Sansom Institute for Health Research Clinical Trial Facility (SIHR CTF) in Adelaide, South Australia. 

### 2.3. Design

The current study employed a 24-week parallel crossover design to compare a Mediterranean diet with 2-3 weekly servings of fresh, lean pork (MedPork) and a low-fat control diet (LF). A low-fat diet was chosen as a comparator for two reasons. Firstly, at the time of study conception, low fat diets were recommended and utilised as a strategy to prevent and treat diet-related chronic disease, such as CVD. Secondly, the current study aimed to replicate the design of the PREDIMED trial, the largest clinical investigation of the Mediterranean diet [24], which also employed a low-fat control diet. Participants were randomised to their first dietary intervention using block randomization, stratified by age and gender. Group 1 (*n* = 16) participants were randomised to complete the MedPork intervention followed by the LF intervention. Group 2 (*n* = 17) participants were randomised to complete the LF intervention followed by the MedPork intervention. The participants followed each diet for 8 weeks, and an 8-week washout period separated interventions. Based on previous nutritional intervention studies, 8 weeks was expected to be an adequate intervention period to detect change in cognitive function [28,29,30]. 

### 2.4. Dietary Interventions

#### 2.4.1. Low-Fat Diet (LF)

Guidelines for the LF diet were based on the PREDIMED study [24]. During the LF intervention, participants were advised to follow their habitual diets, making adjustments to reduce total fat consumption. Participants were instructed to drastically reduce their intake of high-fat foods, such as vegetable oils (≤20mL per day), butter and margarine (≤10mL per day), high-fat meats and dairy, nuts, chocolates, cakes and pastry, and to consume lower fat alternatives. When buying packaged or ready-made foods, participants were advised to select products containing less than 10% of energy from fat, excluding dairy where low-fat milk (<2% fat), yoghurt (<2% fat) and cheese (<25% fat) were recommended. Participants were also instructed to remove visible fat and skin from meat and fish before cooking.

#### 2.4.2. Mediterranean Diet with Pork (MedPork)

During the MedPork intervention, participants were advised to follow a Mediterranean diet while consuming 2–3 weekly serves of fresh, lean pork (see Supplemental Table S1). Mediterranean diet guidelines were adapted from Estruch et al. (2013) [24] for an Australian food supply.

During the MedPork intervention, the following foods were provided each week: 375mL EVOO (donated by Cobram Estate); 250 g of fresh, lean pork; 150 g raw, unsalted almonds (donated by Almond Board of Australia), walnuts and hazelnuts; 225 g (net weight) of canned chickpeas, red kidney beans, 4-bean mix and lentils (donated by Simplot Australia Pty Ltd.); 95 g of canned tuna and 95 g of canned salmon (donated by Simplot Australia Pty Ltd.). 

#### 2.4.3. Dietetic Counselling

At the beginning of each dietary intervention, participants met with a dietitian who delivered dietary education, guidelines and resources [26]. Throughout the dietary intervention, participants also attended bi-weekly dietetic visits to discuss dietary adherence, challenges and adverse effects. 

### 2.5. Measures

#### 2.5.1. Cognitive Function

Cognitive function was assessed using the Cambridge Neuropsychological Test Automated Battery (CANTAB). A battery of tests was selected to measure change across memory, attention, processing speed and planning. These cognitive domains were of particular interest due to their sensitivity to aging, cardiovascular health and nutritional intervention. Supplemental Table S2 details the tests included to assess each cognitive domain. A motor orientation task was performed at the beginning of each testing session, and each subsequent task contained a practice component to familiarise participants with the CANTAB tablet.

Addenbrooke’s Cognitive Examination-Revised (ACE-R) was administered at screening to exclude volunteers with possible mild cognitive impairment (MCI) or dementia [31], and again at Weeks 8 and 24. 

#### 2.5.2. Sleep

The Karolinska Sleep Scale (KSS) was administered prior to each cognitive testing session to gauge the level of sleepiness, which may interfere with cognitive performance. The KSS is a 9-point scale that asked participants to rate their current level of sleepiness where 1 = Extremely alert and 9 = Very sleepy, great effort to keep awake, fighting sleep. If participants scored 6 or above or commented on poor sleep quality of the previous night, cognitive testing was rescheduled. 

#### 2.5.3. Psychological Well-Being

Psychological well-being was evaluated using the SF-36 Health Survey Version II, adapted for use in Australia [32,33], and the Profile of Mood States (POMS) [34]. For measures of psychological well-being, participants were asked to reflect on their perceptions of their health and mood over the previous month.

#### 2.5.4. Dietary Adherence

Adherence was assessed throughout the interventions using diet-specific surveys. A 15-point survey assessed adherence to the MedPork intervention, and a 9-point survey assessed adherence to the LF intervention. Both surveys were modelled on those used in the PREDIMED trial [24]. The surveys were completed by participants every two weeks and returned and scored at dietetic visits. Detailed dietary and nutrient intake was also captured prior to, and at the end of, each intervention using 3-day weighed food records (WFRs). 

### 2.6. Procedure

Participants attended clinic assessment visits at week 0, 8, 16 and 24. One week prior to Week 0, participants also attended a pre-baseline appointment where the study was explained, study resources were provided, and informed consent was obtained. 

The SF-36, POMS and WFRs were completed by participants in the week preceding each assessment visit. To limit the potential for measurement error, cognitive testing was performed under controlled conditions [35]. Participants arrived at clinic assessment visits fasted from food, beverages (excluding water), alcohol and caffeine for 12 h. Following cardiovascular measurements (reported elsewhere), participants consumed a standardised continental breakfast of toast or cereal 15 min prior to cognitive testing. Cognitive testing took place between 9:00 a.m. and 12:00 p.m. in a temperature and noise-controlled environment. CANTAB test administration was standardised through the use of a testing script provided by Cambridge Cognition. 

Participants followed their first intervention between Week 0 and Week 8, and their second intervention between Week 16 and Week 24. During intervention periods, participants also attended bi-weekly dietetic visits (described above). Between Week 8 and Week 16, participants returned to their habitual diet to wash out any effects of the first dietary intervention.

### 2.7. Statistical Analysis

Statistical analyses were conducted using SPSS version 21.0 (SPSS Inc, Chicago, IL, USA) and STATA version 14.2 (StataCorp, College Station, TX, USA). Nutrient intake was calculated using Foodworks 9, databases AusFoods17 and AusBrands17 (Xyris Software Pty Ltd., Spring Hill, Australia).

The current study was powered to detect a clinically significant reduction of 2.5 mmHg in the primary outcome, systolic blood pressure (reported elsewhere) [26]. The study was also powered on major secondary outcomes. To detect a statistically significant difference in cognitive composite scores for attention, processing speed, memory and planning, assuming a correlation between measurements of *r* = 0.6, and an effect size of 0.5 SD units [35,36], a total sample size of *n* = 22 was required for 80% power. 

Cognitive composite scores were calculated by generating z-scores for each CANTAB test. Z-scores were then combined and averaged relative to their cognitive domain: attention, memory, processing speed or planning. For tests where a lower score indicates better performance (i.e., reaction time), raw scores were reversed before being converted to z-scores. 

Baseline characteristics were compared between groups using independent samples t-tests and chi-squared test of independence. Preliminary linear mixed-effects analyses were conducted for all outcomes to detect a significant period and carryover effects. Level 1 and level 2 residuals were tested for normality. Non-normally distributed data was log-transformed using base 10.

The linear mixed-effects models included terms for diet (MedPork vs. LF), Visit (1 or 2), Order (1 or 2) and Period (1 or 2). Visit 1 refers to week 0, and Visit 2 to week 8 of each intervention. Order refers to whether participants followed the MedPork (Order 1) or LF (Order 2) intervention first. Period refers to the phase of the trial in which the intervention was followed: Week 0–8 = Period 1; Week 16–24 = Period 2. A Diet–X-Visit term was included to test the difference in the change between Week 0 and Week 8 between interventions. Treatment effects were estimated using the difference in the adjusted marginal means at Visit 2 and are presented as the estimated mean difference between interventions (MedPork versus LF). Where significant period effects were identified in preliminary analyses, the Diet-X-Visit term was replaced by Diet-X-Visit-X-Period to allow the Diet-X-Visit effect to vary by period. The participant ID number was included in the model as a random intercept. Missing data were accounted for by using the best linear unbiased predictions (BLUP) of the mixed effects models. No adjustments were performed for multiple comparisons in order to preserve the Type II error rate.

## 3. Results

In the current study, 35 participants were enrolled and randomised to their first dietary intervention. Baseline data was collected for 33 participants. A total of 31 participants completed both dietary interventions. 

**Figure 1:**
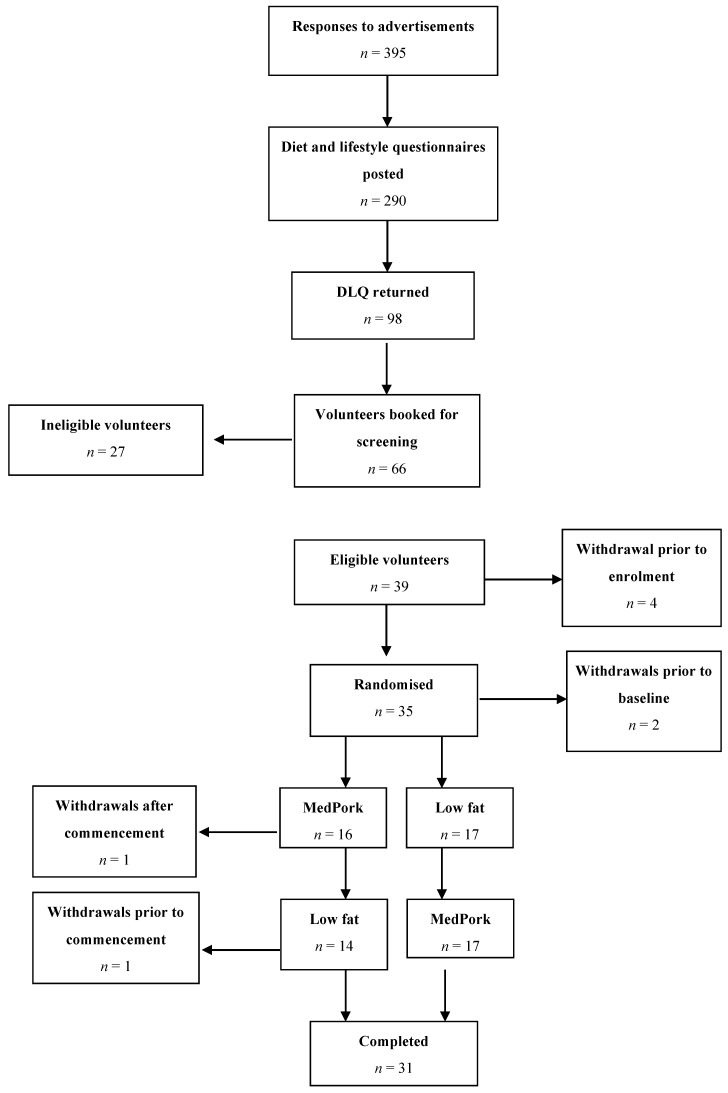
Consort diagram illustrating the flow of the participants from recruitment through to study completion. The intention-to-treat analysis is based on all the participants with baseline data (*n*= 33).

Figure 1 illustrates the Consort flow of the study, detailing time of withdrawals. Following baseline assessments, one participant withdrew during the MedPork intervention in the first period and one participant withdrew during the washout period, prior to commencing the LF intervention. Reasons for withdrawal included competing commitments (*n* = 1) and illness preventing adherence to dietary intervention (*n* = 1). The results below are based on the modified intention to treat (ITT) population (*n* = 33). 

Baseline characteristics of participants are presented in Table 1. A significant difference was detected at baseline for ACE-R score. No other significant differences were observed between groups. 

### 3.1. Period Effects

There were no significant Treatment-X-Visit-X-Period interactions for the cognitive composite scores. However, a significant Treatment–X-Visit-X-Period interaction was found for total mood disturbance using the Profile of Mood States.

### 3.2. Dietary Adherence and Nutrients 

At screening, the mean score for the 15-point Mediterranean diet survey was 6.1 ± 2.1. During the MedPork intervention, the mean score was 13.5 ± 1.7, indicating 90% adherence and an increase of 7.4 points from screening. During the LF intervention, participants achieved a mean score of 8.4 ± 1.1 on the 9-point low-fat survey, indicating 93.3% adherence. 

Nutrient intakes across the intervention periods are presented in Table 2. According to WFRs, participants consumed 0.4 ± 0.1 servings of fresh pork each day, or 3.0 ± 0.7 servings each week, during the MedPork intervention. 

Compared to the LF intervention, the MedPork intervention led to a greater consumption of % energy from fat, % energy from MUFA, % energy from PUFA and MUFA:SFA. The LF intervention led to a significantly higher intake of % energy from protein and carbohydrates. The MedPork intervention led to significantly fewer servings per day of refined grains, and a higher consumption of legumes, seafood, nuts and seeds, EVOO, meat and meat alternatives, but not red meat.

### 3.3. Cognitive Function

The results for cognitive outcomes are presented in Table 3. No significant differences were observed between groups for the cognitive composite scores of memory, attention, planning, or the ACE-R. However, a significant effect was observed for processing speed, where the MedPork intervention was associated with higher performance.

### 3.4. Psychological Well-Being

Results for psychological well-being are reported in Table 4. Compared with the LF intervention, the MedPork intervention led to higher scores for role emotional of the SF-36, with a moderate effect size (*d =* 0.5). No significant differences were reported for the remaining outcomes of the SF-36 Health Survey, nor for the items of the Profile of Mood States. 

## 4. Discussion

The current study aimed to examine the cognitive effects of a Mediterranean diet supplemented with 2–3 weekly servings of fresh, lean pork. During the MedPork intervention, participants achieved high adherence to the Mediterranean diet while consuming an average of 3 servings of fresh, lean pork each day. Further, participants consumed significantly more legumes, nuts and seeds, seafood and extra virgin olive oil. Compared with the LF control diet, the MedPork intervention led to higher performance in the cognitive domain of processing speed and higher scores for the SF-36 subscale emotional role functioning. No significant differences were observed for other domains of cognitive function, nor for additional measures of psychological well-being.

Processing speed is most commonly indexed by simple and choice reaction time. Processing speed is a marker of brain connectivity, and is integral to the coordination of higher order functions [37,38]. Further, processing speed is an indicator of cognitive aging and is a potential mediator of age-related change across other cognitive functions [39]. Salthouse et al. proposed that changes in processing speed could be partially responsible for age-related decline across cognitive processes that rely on processing speed, such as memory [40,41]. Additionally, slowing in processing speed is an indicator of mild cognitive impairment and dementia [42]. 

A recent publication of normative CANTAB data confirmed a positive relationship between processing speed and age, with an increase of 0.6 ms per year in simple reaction time and an increase of 0.9 ms per year for choice reaction time [43]. Improvements in processing speed may be capable of attenuating these age-related declines. It is also possible that improvements to processing speed could lead to future improvements across other cognitive domains [40,41]. Although promising, the variability of our data must be acknowledged. Normative CANTAB data indicates a standard deviation of ±43 ms for simple and choice reaction tests [43]. Reflected in our own results, this high level of variability in treatment effects between subjects reduced our observed effect size. Further investigation of the long-term effects of a Mediterranean diet on processing speed is therefore warranted.

The findings of the current study are consistent with those of a previous investigation conducted by our research group. In the MedDairy trial, a Mediterranean diet supplemented with 3 daily servings of dairy foods led to higher processing speed performance and mood scores over 8 weeks, compared with a low-fat control [9]. This suggests that our positive cognitive findings are likely due to components of the Mediterranean diet, rather than the addition of fresh pork. Few studies have examined the association between pork and cognitive function. However, a pilot study conducted in older, community dwelling adults reported that substituting chicken with pork did not have adverse effects on cognitive function [18]. This is supported by the current study, which found positive cognitive outcomes consistent with the MedDairy trial.

Observational research has indicated that the Mediterranean diet may be capable of improving cognitive function to reduce cognitive decline and delay, or even prevent, the onset of dementia. However, a recent meta-analysis reported that results from RCTs were predominantly non-significant, with small effect sizes [44]. The lack of consistency across studies has been attributed to varied methodologies and cognitive testing measures. Of the five studies included in the meta-analysis, the robustly designed and long term PREDIMED trial (*n* = 1497) reported the most convincing results. Participants following Mediterranean dietary interventions achieved greater scores on cognitive tests of global cognition, memory and attention, compared with controls [8]. The current study design was modelled on the PREDIMED trial, but did not detect changes across attention and memory. This may be due to the use of different cognitive measures, although our battery of neuropsychological tests intended to capture the same cognitive constructs as the PREDIMED trial [26]. Therefore, it is believed that this study’s inability to elicit results similar to those observed in the PREDIMED trial may be due the study’s duration. Specifically, the improvements observed in memory function in the PREDIMED trial were observed over years, as opposed to weeks. 

Notably, all five studies in the meta-analysis conducted by Radd-Vagenas et al. (2018) [44] examined processing speed. However, no study found significant effects of the Mediterranean diet. The MedPork trial was therefore one of the first to identify significant associations between the Mediterranean diet and processing speed. The ability of the MedPork trial to have captured these changes could be due to the use of computerized testing, which increased the accuracy of detecting changes measured in milliseconds.

As reported elsewhere [23], this study did not detect significant effects in cardiovascular health following the MedPork intervention. Our findings therefore support the hypothesis that nutrients contained within the Mediterranean diet influenced brain function directly, independent of vascular change. For example, mono and polyunsaturated fatty acids have been identified as neuroprotective. Specifically, long-chain omega-3 fatty acids have been found to improve white matter integrity and processing speed [45]. Further, flavonoids and carotenoids contained within the Mediterranean diet have been found to have antioxidant effects to preserve neural cell integrity and function, while vitamin B12, vitamin E and folate have been found to protect against cognitive decline [29,46]. 

Our study also detected a significant effect of diet on emotional role functioning, a subscale of the mental health measure assessed by the SF-36 Health Survey. Emotional role functioning encompasses issues experienced in daily life due to emotional problems. Items in the emotional role functioning subscale focus on whether anxiety and depression influence perceptions of work and other activities [33]. No other significant effects were found for measures of psychological well-being, although subscales of the POMS were higher following the MedPork intervention. Notably, participant scores on the SF-36 and POMS were within one standard deviation of the population norms. This indicates that our sample was relatively healthy, and was thus one which may not respond to the psychological effects of the diet Notwithstanding, our findings concerning emotional role functioning are consistent with previous literature reporting positive associations between Mediterranean diet adherence and quality of life in adolescent and middle-aged populations [47,48,49,50]. Further, intervention trials have reported positive effects of the Mediterranean diet on measures of depression in middle-aged and clinical samples [9,51,52]. As depression is a leading risk factor for dementia, future research should continue this line of enquiry, especially in older age groups [2].

Inflammation has been implicated in the pathology of depression [53]. It has been proposed previously that improvements to mood are due to the anti-inflammatory components of the Mediterranean diet, such as omega-3 fatty acids [51]. Similarly, healthful dietary patterns have been linked to improved wellbeing and a reduced risk of depression [54]. In comparison to baseline consumption, both dietary interventions led to an increase in the consumption of fruits, vegetables and whole grains, and reduced consumption of saturated fat. These improvements to overall diet quality may explain the trends of higher mood and well-being scores observed across both dietary interventions. 

The current study study has demonstrated that an Australian sample is capable of achieving high adherence to a Mediterranean diet supplemented with fresh lean pork. High dietary adherence was also observed during the LF intervention. High compliance to each intervention was likely influenced by regular dietetic visits and the provision of food supplies during the MedPork phase. Future studies are therefore required to determine longer-term adherence in non-clinical settings.

The MedPork trial had a number of key strengths. A parallel cross-over design was chosen to increase the power and limit the influence of any potential differences in subject characteristics. Dietetic counselling was delivered to promote participant understanding of, and adherence to dietary interventions. The authors performed a comprehensive battery of cognitive tests and used a computerised mode of cognitive testing delivered in a standardised testing environment to reduce measurement error. The MedPork trial was also one of the first to explore the cognitive effects of pork as part of a healthy dietary pattern.

The following limitations should also be considered. Firstly, the use of a low-fat control diet limits the ability to appraise the differences between a Mediterranean diet supplemented with pork and a traditional Mediterranean diet. Further, the majority of Australians do not follow a low-fat diet. Therefore, it is unknown whether similar effects would be seen in comparison with a typical Australian diet. It is also possible that our cognitive findings were due to a sub-optimal effect of the low-fat diet on brain function, as opposed to a beneficial effect of the Mediterranean diet. However, a randomised controlled trial (RCT) of adults aged between 28 and 64 years found no significant effect of a low-fat diet on processing speed over a period of 12 months [20]. Although our study was powered to detect change in cognitive outcomes, the small sample size may not be reflective of the wider population which could reduce the generalizability of our results. Further, the study duration may have limited the capacity to detect a change in cognitive function, given that cognitive changes are likely to occur over a number of years. Similarly, participant ages ranged between 45 and 80 years. While the sample were at risk of CVD and therefore dementia, cognitive change may be less likely in younger participants who have not yet experienced age-related cognitive decline. Likewise, individuals with evidence of cognitive impairment, in whom greater cognitive change may have been observed, were excluded. Finally, several measures, including WFRs and psychological instruments, relied on self-reporting, which is susceptible to inaccuracies. While controlled-feeding studies offer greater accuracy, such a controlled environment cannot be generalised to the wider population. The current study therefore employed WFRs, the gold standard of dietary assessment, to gain comprehensive dietary intake data. Likewise, the SF-36 and POMS were chosen due to their high validity and reliability.

The current study aimed to examine the cognitive effects of a Mediterranean diet with 2–3 weekly servings of fresh lean pork. Our findings indicate that the MedPork intervention was capable of improving processing speed and mood in a population at risk of CVD and therefore dementia. Our findings provided further support for the cognitive effects of the Mediterranean diet, and the pathway through which the diet may influence cognitive function. 

## Figures and Tables

**Table 1:** The demographic and clinical characteristics of the study sample at baseline, according to the first dietary intervention ^1^.

	Total (*n* = 33)	Group 1 (*n* = 16)	Group 2 (*n* = 17)
Age (years)	61.0 ± 7.1	60.2 ± 8.7	61.6 ± 5.7
Gender			
Males (%)	10 (30.3)	6 (40.0)	4 (22.2)
Females (%)	23 (69.7)	9 (60.0)	14 (77.8)
Education (years)	16.7 ± 4.4	15.7 ± 3.5	17.5 ± 4.9
Home SBP (mmHg)	128.9 ± 12.1	129.2 ± 14.0	128.5 ± 10.4
BMI (kg/m^2^)	30.6 ± 5.1	31.6 ± 5.7	29.8 ± 4.6
ACE-R	95.1 ± 3.8	93.7 ± 4.2	96.4 ± 2.8 *

^1^ Values are presented as mean ± standard deviation Between group differences compared using independent samples t-tests and chi-squared test of independence. Group 1 received MedPork intervention first; Group 2 received LF intervention first; SBP, systolic blood pressure, BMI, body mass index; ACE-R, Addenbrooke’s Cognitive Examination-Revised. * Significant difference between groups at *p* < 0.05.

**Table 2:** Energy and nutrient intakes at baseline and week 8, including between group differences (*n* = 33) ^1.^

	MedPork Diet				LF Diet				Estimated Mean Difference between Interventions (MedPork vs LF) at 8 Weeks ^2^	*p* ^3^
Nutrients	Baseline		Week 8		Baseline		Week 8			
	Mean	SE	Mean	SE	Mean	SE	Mean	SE	(95% CI)	
Energy (MJ/day)	8.96 ± 0.33		8.33 ± 0.33		9.00 ± 0.33		7.86 ± 0.34		0.47 (−0.32, 1.27)	0.24
% en from protein	18.40 ± 0.65		19.71 ± 0.67		19.45 ± 0.66		22.21 ± 0.68		−2.51 (−4.10, 0.91)	<0.01
% en from total fat	34.08 ± 1.25		36.84 ± 0.67		34.59 ± 1.27		27.52 ± 1.31		9.32 (5.91, 12.74)	<0.001
% en from SFA	12.07 ± 0.57		9.18 ± 0.58		12.24 ± 0.57		9.23 ± 0.59		−0.05 (−1.41, 1.31)	0.94
% en from MUFA	5.41 ± 0.34		7.61 ± 0.35		5.53 ± 0.35		5.05 ± 0.36		2.56 (1.61, 3.51)	<0.001
% en from PUFA	13.89 ± 0.76		17.37 ± 0.79		14.16 ± 0.077		10.68 ± 0.80		6.69 (4.55, 8.83)	<0.001
MUFA:SFA ^4^	1.24 ± 0.09		1.99 ± 0.09		1.19 ± 0.09		1.18 ± 0.09		1.69 (1.46, 1.95)	<0.001
% en from CHO	39.50 ± 1.37		35.23 ± 1.37		38.59 ± 1.35		41.23 ± 1.39		−6.00 (−9.22, −2.79)	<0.001
% en from alcohol	4.41 ± 0.81		3.45 ± 0.82		3.54 ± 0.82		3.99 ± 0.83		−0.55 (−1.75, 0.65)	0.37
Cholesterol (mg/MJ) ^4^	37.76 ± 0.34		33.64 ± 3.48		43.57 ± 3.44		38.97 ± 3.58		0.82 (0.68, 1.01)	0.06
Fibre (g/MJ)	3.03 ± 0.19		3.98 ± 0.19		3.06 ± 0.19		3.84 ± 0.20		0.14 (−0.31, 0.59)	0.54
Vitamin C (mg/MJ) ^4^	11.34 ± 0.39		21.53 ± 1.56		12.53 ± 1.51		17.26 ± 1.58		0.81 (0.96, 1.59)	0.10
Vitamin E (mg/MJ) ^4^	1.64 ± 0.11		2.14 ± 0.12		1.47 ± 0.12		1.40 ± 0.12		1.57 (1.33, 1.85)	<0.001
Total vit A equiv. (mg/MJ) ^4^	0.11 ± 0.01		0.13 ± 0.01		0.12 ± 0.01		0.12 ± 0.01		0.01(−0.01, 0.01)	0.96
Total folate (µg/MJ)	61.65 ± 3.56		68.28 ± 3.68		63.94 ± 3.63		81.11 ± 3.74		−12.83 (−21.57, 4.09)	<0.01
β-carotene equiv. (mg/MJ) ^4^	0.49 ± 0.08		0.67 ± 0.08		0.51 ± 0.08		0.58 ± 0.08		0.01 (−0.01, 0.01)	0.45
Sodium (g/MJ)	0.28 ± 0.02		0.24 ± 0.02		0.32 ± 0.02		0.28 ± 0.02		−0.04 (−0.08, 0.01)	0.06
Calcium (g/MJ)	0.10 ± 0.01		0.11 ± 0.01		0.11 ± 0.01		0.12 ± 0.01		−0.01 (−0.02, 0.01)	0.14
Iron (mg/MJ) ^4^	1.48 ± 0.07		1.48 ± 0.07		1.39 ± 0.07		1.63 ± 0.07		0.93 (0.82, 1.05)	0.21
Zinc (mg/MJ)	1.32 ± 0.06		1.27 ± 0.06		1.30 ± 0.06		1.40 ± 0.06		−0.12 (−0.26, 0.01)	0.07
Linoleic acid (g/MJ)	1.18 ± 0.08		1.69 ± 0.08		1.23 ± 0.08		1.11 ± 0.09		0.59 (0.36, 0.81)	<0.001
α-linolenic acid (g/MJ) ^4^	0.17 ± 0.01		0.23 ± 0.02		0.16 ± 0.02		0.16 ± 0.02		1.49 (1.19, 1.86)	<0.001
**Servings/day**										
Whole grains ^4^	2.14 ± 0.26		2.44 ± 0.27		1.70 ± 0.26		2.03 ± 0.27		1.49 (0.88, 2.54)	0.14
Refined grains ^4^	3.61 ± 0.37		1.96 ± 0.38		3.84 ± 0.38		3.45 ± 0.38		0.65 (0.49, 0.85)	<0.01
Fruits	1.30 ± 0.17		2.20 ± 0.18		1.57 ± 0.18		1.74 ± 0.18		0.46 (0.05, 0.86)	0.03
Vegetables ^4^	4.39 ± 0.60		5.83 ± 0.62		3.66 ± 0.61		5.02 ± 0.64		1.25 (0.95, 1.62)	0.10
Legumes ^4^	0.19 ± 0.09		0.63 ± 0.09		0.23 ± 0.09		0.35 ± 0.09		1.90 (1.19, 3.04)	<0.01
Meat/meat altern.	2.71 ± 0.19		3.43 ± 0.20		2.95 ± 0.20		2.62 ± 0.20		0.81 (0.34, 1.27)	<0.001
Red meat ^4^	0.64 ± 0.13		0.85 ± 0.13		0.78 ± 0.13		0.71 ± 0.13		1.09 (0.95, 1.25)	0.24
Fresh pork ^4^	0.09 ± 0.05		0.44 ± 0.06		0.16 ± 0.05		0.13 ± 0.05		1.13 (1.06, 1.20)	<0.001
Seafood ^4^	0.38 ± 0.08		0.64 ± 0.08		0.30 ± 0.08		0.39 ± 0.08		1.19 (1.05, 1.36)	<0.01
Nuts and seeds ^4^	0.47 ± 0.08		0.96 ± 0.08		0.50 ± 0.08		0.22 ± 0.08		1.61 (1.42, 1.84)	<0.001
Total dairy ^4^	1.80 ± 0.20		1.60 ± 0.20		1.92 ± 0.20		1.93 ± 0.20		0.85 (0.64, 1.11)	0.23
EVOO (tsp/day) ^4^	1.14 ± 0.39		4.51 ± 0.39		1.15 ± 0.39		0.68 ± 0.39		2.83 (2.20, 3.65)	<0.001

^1^ Values are mean ± SE. ^2^ Estimated marginal mean difference from linear mixed effects models. Differences between interventions were analysed by linear mixed effects models, including fixed effect terms for Group, Visit, Group X Visit, Period, Order and Weight (excluding weight-related variables). ^3^ Adjusted for differences between treatments. ^4^ Transformed variable: Observed mean ± SE are presented together with rate ratio change in the geometric mean and associated *p*-value. En, energy; SFA, saturated fat; MUFA, monounsaturated fat; PUFA, polyunsaturated fat; CHO, carbohydrates, Vit, vitamin; equiv, equivalents; altern, alternatives.

**Table 3:** The standardised cognitive composite scores and ACE-R at the baseline and final visit, including between group differences (*n* = 33) ^1^.

	MedPork Diet				LF Diet				Estimated Mean Difference between Interventions (MedPork vs LF) at 8 Weeks ^2^	*p* ^3^
	Baseline		Week 8		Baseline		Week 8			
	Mean	SE	Mean	SE	Mean	SE	Mean	SE	(95% CI)	
Attention	−0.14 ± 0.11		0.17 ± 0.11		−0.06 ± 0.11		0.14 ± 0.11		0.04 (−0.14, 0.21)	0.70
Processing speed	−0.04 ± 0.16		0.13 ± 0.16		0.01 ± 0.16		−0.19 ± 0.16		0.32 (0.08, 0.57)	0.01
Memory	0.04 ± 0.09		-0.08 ± 0.09		0.03 ± 0.09		0.07 ± 0.09		−0.15 (−0.31, 0.01)	0.06
Planning	−0.18 ± 0.11		0.15 ± 0.11		−0.05 ± 0.11		0.09 ± 0.11		0.06 (−0.14, 0.26)	0.56
ACE-R	95.98 ± 0.52		96.19 ± 0.53		95.13 ± 0.52		96.37 ± 0.53		−0.18 (−1.12, 0.76)	0.71

^1^ Values are mean ± SE. ^2^ Estimated marginal mean difference from linear mixed effects models ^3^ Adjusted for baseline differences between treatments.

**Table 4:** SF-36 Health Survey and Profile of Mood States at baseline and week 8, including between group differences (*n* = 33) ^1^.

		MedPork Diet				LF Diet				Estimated Mean Difference between Interventions (MedPork vs LF) at 8 Weeks ^3^	*p* ^4^
	Normative Data ^2^	Baseline		Week 8		Baseline		Week 8			
	Mean (SD)	Mean + SE		Mean + SE		Mean + SE		Mean + SE		(95% CI)	
SF-36 Health Survey											
Physical functioning ^5^	92.5 (13.4)	81.57 ± 2.88		85.82 ± 2.96		83.56 ± 2.96		83.47 ± 2.95		0.95 (0.64, 1.38)	0.80
Role physical ^5^	91.4 (23.2)	84.58 ± 3.19		91.54 ± 3.35		84.88 ± 3.13		88.95 ± 3.33		0.71 (0.38, 1.32)	0.27
Bodily pain	86.3 (17.9)	71.54 ± 3.36		78.66 ± 3.47		75.72 ± 3.33		74.74 ± 3.47		3.92 (−1.84, 9.67)	0.18
General health	78.8 (15.7)	68.84 ± 3.39		74.18 ± 3.46		71.12 ± 3.38		74.99 ± 3.45		−0.80 (−5.14, 3.54)	0.72
Vitality	64.0 (18.2)	61.45 ± 3.05		65.51 ± 3.19		64.11 ± 3.02		67.89 ±3.18		−2.37 (−8.54, 3.79)	0.45
Social functioning ^5^	91.3 (15.8)	89.96 ± 2.80		89.93 ± 2.97		89.64 ± 2.73		91.71 ± 2.95		1.05 (0.56, 2.00)	0.87
Role emotional ^5^	85.6 (29.3)	93.26 ± 2.22		96.87 ± 2.33		91.67 ± 2.18		91.30 ± 2.32		0.55 (0.32, 1.05)	0.03
Mental health ^5^	75.4 (16.3)	82.97 ±1.96		85.80 ±2.01		83.70 ±1.92		83.61 ±1.98		0.83 (0.66, 1.05)	0.10
Physical component score	-	49.60 +1.34		52.27 + 1.36		50.98 + 1.34		51.61 + 1.37		0.65 (−1.23, 2.54)	0.47
Mental component score	-	54.92 + 1.08		55.68 + 1.11		55.00 + 1.08		54.40 + 1.12		1.29 (−0.63, 3.20)	0.19
POMS											
Tension ^5^	7.0 (5.5)	5.27 ±0.75		4.54 ±0.76		5.29 ±0.76		5.20 ±0.76		0.87 (0.71, 1.07)	0.20
Depression ^5^	7.1 (8.4)	3.50 ± 0.63		2.36 ± 0.64		3.21 ± 0.63		3.59 ± 0.63		0.77 (0.58, 1.02)	0.08
Anger ^5^	6.6 (6.7)	3.81 ± 0.67		3.01 ± 0.67		3.06 ± 0.67		3.71 ± 0.67		0.85 (0.68, 1.10)	0.20
Confusion ^5^	5.2 (4.1)	4.34 ± 0.57		3.97 ± 0.58		4.21 ± 0.58		3.89 ± 0.59		1.10 (0.91, 1.29)	0.36
Fatigue ^5^	7.3 (5.7)	5.85 ± 0.75		5.14 ± 0.77		6.09 ± 0.77		6.53 ± 0.77		0.87 (0.68, 1.12)	0.31
Vigour	20.2 (6.2)	18.36 ± 1.11		19.15 ± 0.12		17.90 ± 1.12		18.12 ± 1.12		1.03 (−0.23, 2.28)	0.11
Total Mood Disturbance ^5^	12.7 (29.6)	5.81 ±0.32		5.41 ±0.32		5.77 ±0.32		5.80 ±0.32		0.87 (0.72, 1.05)	0.12

^1^ Values are mean ± SE., excluding norms which are presented as mean (SD) ^2^ Normative values of SF-36 are based on healthy adults aged 18-64 years [6]; normative values of POMS are based on healthy adults aged ≥25 years [7], ^3^ Estimated marginal mean difference from linear mixed effects models, ^4^ Adjusted for differences between treatments, ^5^ Transformed variable: Observed mean ± SE are presented together with rate ratio change in the geometric mean and associated *p*-value.

## Supplementary Materials

The following are available online at https://www.mdpi.com/2072-6643/11/7/1521/s1, Table S1. MedPork dietary guidelines; Table S2. Composition of cognitive composite scores.
