# Supplementary material for: A Mediterranean Diet with Fresh, Lean Pork Improves Processing Speed and Mood: Cognitive Findings from the MedPork Randomised Controlled Trial
Source: Nutrients. 2019 Jul 4;11(7):1521. doi: 10.3390/nu11071521 (PMC6683093; doi:10.3390/nu11071521)
Supplement: Supplementary file 1 [file nutrients-11-01521-s001.pdf]

Table S1. MedPork dietary guidelines.

Consume every day	• Minimum of 20 mL of extra virgin olive oil (EVOO);<br>• ≥3-4 servings of fresh vegetables<br>• ≥2–3 servings of fresh fruit (one serving = 150g fresh, 30g dried, or 250mL canned in natural juice);<br>• For usual drinkers, red wine is recommended as the main source of alcohol with a maximum of two standard drinks per day (10g ethanol = 1 standard drink) [47]
Consume every week	• ≥3 servings of legumes (one serving = 75g);<br>• 2-3 servings of fresh, lean pork (one serving = 100g cooked)<br>• ≥3 servings of fish and seafood (at least one serving of oily fish) (one serving = 100g cooked);<br>• ≥5 servings of raw or roasted nuts or seeds, without added salt, sugar or chocolate (one serving = 30g; 7.5g hazelnuts, 15g walnuts, 7.5g almonds supplied for each serving);<br>• Cook regularly (at least twice a week) with a tomato-based sauce (EVOO, tomato, garlic and onion);<br>• Limit consumption of red and cured meat (remove all visible fat) to ≤1 serving/week (one serving of red meat/cured ham = 100g);<br>• Replace chicken and red meat with pork, and ensure that total weekly meat consumption did not exceed 400g (4 servings)<br>• Limit consumption of chocolate to ≤1 serving/week (one serving of chocolate = 50g);
General guidelines	• Ad-libitum consumption of wholegrain cereal products (bread, pasta, rice, cereal) and dairy

- Select white meats (poultry without skin) instead of red meats or processed meats;
- Eliminate or limit the consumption of cream, butter, margarine, cold meat, pate, duck, carbonated and or sugared beverages, pastries, commercial bakery products (cakes, donuts, cookies), desserts (puddings), French fries, potato crisps, sweets;

Table S2. Composition of cognitive composite scores.

Composite Score	CANTAB test and measure
Attention	AST latency correct (ms) [a]
	AST correct (%)
	RVP A
Processing speed	Simple RT (ms) [a]
	Choice RT (ms) [a]
Memory	DMS total correct
	PAL total errors adjusted [a]
	SWM total errors [a]
	VRM recall
	VRM recognition
Planning	OTS problems solved first choice
	OTS choice to correct [a]
	OTS latency correct (sec) [a]
	SWM Strategy [a]

CANTAB, Cambridge Neuropsychological Automated Test Battery; AST, Attention Switching Task; DMS, Delayed Match to Sample; OTS, One Touch Stockings of Cambridge; PAL, Paired Associates Learning; RT, Reaction Time; RVP, Rapid Visual Processing; SWM, Spatial Working Memory; VRM, Verbal Recognition Memory. [a] Raw score reversed for calculation of composite score
